# Supplementary material for: Modeling of the Spatial Distribution of Forest Carbon Storage in a Tropical/Subtropical Island with Multiple Ecozones
Source: Plants (Basel). 2023 Jul 26;12(15):2777. doi: 10.3390/plants12152777 (PMC10421002; doi:10.3390/plants12152777)
Supplement: Supplementary file 1 [file plants-12-02777-s001.zip › plants-2491022-supplementary.pdf]

Supplementary of

Modeling for the spatial distribution of forest carbon storage in a tropical/subtropical island with multiple ecozones

Ting-Wei Chang [1], Guan-Fu Chen [2] and Ken-Hui Chang [2,*]

[1] Department of Environmental and Life Sciences, University of Shizuoka, 52-1 Yada, Suruga Dist., Shizuoka 422-8526, Japan
[2] Department of Safety, Health and Environmental Engineering, National Yunlin University of Science & Technology, 123 University Road, Section 3, Douliu, Yunlin 64002, Taiwan
* Correspondence: ken@airlab.yuntech.edu.tw

Table S1 Stock volume equation of each plant species respectively at respective ecozones

Forest type	Abbreviation	Referred species	Volume equation (Subtropical mountain)	Volume equation (Subtropical humid forest)	Volume equation (Tropical rainforest)	Tropical dry forest	Tropical moist deciduous forest
Natural Fir Forest	FIR-NF	*Abies kawakamii*	$V=-0.5066+0.005367\times DBH+0.000696\times DBH^2$	$V=-0.694737+0.006194\times DBH+0.000834\times DBH^2$	$V=-0.735678+0.006270\times DBH+0.000828\times DBH^2$	$V=-0.694737+0.006194\times DBH+0.000834\times DBH^2$	$V=-0.735678+0.006270\times DBH+0.000828\times DBH^2$
Natural Tsuga Forest	TSU-NF	*Tsuga chinensis*	$V=-0.1795-0.004396\times DBH+0.000695\times DBH^2$	$V=0.7847-0.03527\times DBH+0.00088\times DBH^2$	$V=0.7295-0.035418\times DBH+0.0008846\times DBH^2$	$V=0.7847-0.03527\times DBH+0.00088\times DBH^2$	$V=0.7295-0.035418\times DBH+0.0008846\times DBH^2$
Natural Cypress Forest	CYP-NF	*Chamaecyparis taiwanensis*	$V=1.1439-0.0455\times DBH+0.0014\times DBH^2$	$V=1.1439-0.0455\times DBH+0.0014\times DBH^2$	$V=0.0000944\times DBH^{1.9947405}\times H^{0.659691}$	$V=0.0000944\times DBH^{1.9947405}\times H^{0.659691}$	$V=0.0000944\times DBH^{1.9947405}\times H^{0.659691}$
		Chamaecyparis formosensis	$V=0.00010092\times DBH^{1.54106}\times H^{1.155141}$	$V=0.00010092\times DBH^{1.54106}\times H^{1.155141}$	$V=-0.00324-0.00022\times DBH+0.00090\times H+0.00004\times DBH^2\times H-0.00001\times H^2$	$V=-0.00324-0.00022\times DBH+0.00090\times H+0.00004\times DBH^2\times H-0.00001\times H^2$	$V=-0.00324-0.00022\times DBH+0.00090\times H+0.00004\times DBH^2\times H-0.00001\times H^2$
Natural Pine Forest	PIN-NF	*Pinus taiwanensis*	$V=0.0001547675\times DBH^{1.700988}\times H^{0.721114}$	$V=0.0001547675\times DBH^{1.700988}\times H^{0.721114}$	$V=0.0001547675\times DBH^{1.700988}\times H^{0.721114}$	$V=0.0001547675\times DBH^{1.700988}\times H^{0.721114}$	$V=0.0001547675\times DBH^{1.700988}\times H^{0.721114}$
Natural Spruce Forest	SPR-NF	*Picea morrisonicola*	$V=(-1.0731)+0.021053\times DBH+0.000797\times DBH^2$	$V=(-1.0731)+0.021053\times DBH+0.000797\times DBH^2$	$V=-0.1997-0.02171\times DBH+0.000783\times DBH^2$	$V=-1.0731+0.021053\times DBH+0.000797\times DBH^2$	$V=-0.1997-0.02171\times DBH+0.000783\times DBH^2$
Other Natural Coniferous Forest	O-C-NF	Mixed Conifer	$V=0.0000625\times DBH^{1.77924}\times H^{1.05866}$	$V=0.0000625\times DBH^{1.77924}\times H^{1.05866}$	$V=0.0000625\times DBH^{1.77924}\times H^{1.05866}$	$V=0.0000625\times DBH^{1.77924}\times H^{1.05866}$	$V=0.0000625\times DBH^{1.77924}\times H^{1.05866}$
Cypress Plantation	CYP-P	*Chamaecyparis taiwanensis*	$V=1.1439-0.0455\times DBH+0.0014\times DBH^2$	$V=1.1439-0.0455\times DBH+0.0014\times DBH^2$	$V=0.0000944\times DBH^{1.9947405}\times H^{0.659691}$	$V=0.0000944\times DBH^{1.9947405}\times H^{0.659691}$	$V=0.0000944\times DBH^{1.9947405}\times H^{0.659691}$
Pine Plantation	PIN-P	*Pinus massoniana*	$V=0.0000625\times DBH^{1.77924}\times H^{1.05866}$	$V=0.0000625\times DBH^{1.77924}\times H^{1.05866}$	$V=0.0000625\times DBH^{1.77924}\times H^{1.05866}$	$V=0.0000625\times DBH^{1.77924}\times H^{1.05866}$	$V=0.0000625\times DBH^{1.77924}\times H^{1.05866}$
Luanta Fir Plantation	LF-P	*Cunninghamia konishii*	$V=0.0000702\times DBH^{1.8942224}\times H^{0.8869654}$	$V=0.0000702\times DBH^{1.8942224}\times H^{0.8869654}$	$V=0.0000702\times DBH^{1.8942224}\times H^{0.8869654}$	$V=0.0000702\times DBH^{1.8942224}\times H^{0.8869654}$	$V=0.0000702\times DBH^{1.8942224}\times H^{0.8869654}$
Taiwania Plantation	TAI-P	*Taiwania cryptomerioides*	$V=0.0000944\times DBH^{1.9947405}\times H^{0.659691}$	$V=0.0000944\times DBH^{1.9947405}\times H^{0.659691}$	$V=0.0000944\times DBH^{1.9947405}\times H^{0.659691}$	$V=0.0000944\times DBH^{1.9947405}\times H^{0.659691}$	$V=0.0000944\times DBH^{1.9947405}\times H^{0.659691}$
Japanese Cedar Plantation	JC-P	*Cryptomeria japonica*	$V=17^{-4.193148}\times DBH^{1.867658}\times H^{0.933828}$	$V=0.0000597663\times DBH^{1.867658}\times H^{0.974340}$	$V=17^{-4.193148}\times DBH^{1.867658}\times H^{0.974340}$	$V=17^{-4.193148}\times DBH^{1.867658}\times H^{0.974340}$	$V=17^{-4.193148}\times DBH^{1.867658}\times H^{0.974340}$
Taiwan Incense Cedar Plantation	TIC-P	*Calocedrus formosana*	$V=0.0000944\times DBH^{1.9947405}\times H^{0.659691}$	$V=0.0000944\times DBH^{1.9947405}\times H^{0.659691}$	$V=0.0000944\times DBH^{1.9947405}\times H^{0.659691}$	$V=0.0000944\times DBH^{1.9947405}\times H^{0.659691}$	$V=0.0000944\times DBH^{1.9947405}\times H^{0.659691}$
Other Conifer Plantation	O-C-P	Mixed Conifer	$V=0.0000625\times DBH^{1.77924}\times H^{1.05866}$	$V=0.0000625\times DBH^{1.77924}\times H^{1.05866}$	$V=0.0000625\times DBH^{1.77924}\times H^{1.05866}$	$V=0.0000625\times DBH^{1.77924}\times H^{1.05866}$	$V=0.0000625\times DBH^{1.77924}\times H^{1.05866}$
Mixed Conifer Plantation	MC-P	Mixed Conifer	$V=0.0000625\times DBH^{1.77924}\times H^{1.05866}$	$V=0.0000625\times DBH^{1.77924}\times H^{1.05866}$	$V=0.0000625\times DBH^{1.77924}\times H^{1.05866}$	$V=0.0000625\times DBH^{1.77924}\times H^{1.05866}$	$V=0.0000625\times DBH^{1.77924}\times H^{1.05866}$
Private Coniferous Plantation	P-C-P	Mixed Broadleaf	$V=0.0000464\times DBH^{1.53578}\times H^{1.50657}$	$V=0.0000464\times DBH^{1.53578}\times H^{1.50657}$	$V=0.0000464\times DBH^{1.53578}\times H^{1.50657}$	$V=0.0000464\times DBH^{1.53578}\times H^{1.50657}$	$V=0.0000464\times DBH^{1.53578}\times H^{1.50657}$
New Coniferous Plantation	NEW-C-P	Mixed Conifer	$V=0.0000625\times DBH^{1.77924}\times H^{1.05866}$	$V=0.0000625\times DBH^{1.77924}\times H^{1.05866}$	$V=0.0000625\times DBH^{1.77924}\times H^{1.05866}$	$V=0.0000625\times DBH^{1.77924}\times H^{1.05866}$	$V=0.0000625\times DBH^{1.77924}\times H^{1.05866}$
Natural Broadleaved Forest	B-NF	Mixed Broadleaf	$V=0.0000464\times DBH^{1.53578}\times H^{1.50657}$	$V=0.0000464\times DBH^{1.53578}\times H^{1.50657}$	$V=0.0000464\times DBH^{1.53578}\times H^{1.50657}$	$V=0.0000464\times DBH^{1.53578}\times H^{1.50657}$	$V=0.0000464\times DBH^{1.53578}\times H^{1.50657}$
Natural Mixed Broadleaved Forest	MB-NF	Mixed Broadleaf	$V=0.0000464\times DBH^{1.53578}\times H^{1.50657}$	$V=0.0000464\times DBH^{1.53578}\times H^{1.50657}$	$V=0.0000464\times DBH^{1.53578}\times H^{1.50657}$	$V=0.0000464\times DBH^{1.53578}\times H^{1.50657}$	$V=0.0000464\times DBH^{1.53578}\times H^{1.50657}$
Acacia Plantation	ACA-P	*Acacia confusa*	$V=0.0002045\times DBH^{1.4366684}\times H^{0.8480426}$	$V=0.0002045\times DBH^{1.4366684}\times H^{0.8480426}$	$V=0.0002045\times DBH^{1.4366684}\times H^{0.8480426}$	$V=0.0002045\times DBH^{1.4366684}\times H^{0.8480426}$	$V=0.0002045\times DBH^{1.4366684}\times H^{0.8480426}$
Sweet Gum Plantation	SG-P	*Liquidambar formosana*	$V=0.0000834\times DBH^{1.8761885}\times H^{0.8058127}$	$V=0.0000834\times DBH^{1.8761885}\times H^{0.8058127}$	$V=0.0000834\times DBH^{1.8761885}\times H^{0.8058127}$	$V=0.0000834\times DBH^{1.8761885}\times H^{0.8058127}$	$V=0.0000834\times DBH^{1.8761885}\times H^{0.8058127}$
Camphor Plantation	CAM-P	*Cinnamomum camphora*	$V=0.0000489823\times DBH^{1.60450}\times H^{1.25502}$	$V=0.0000489823\times DBH^{1.60450}\times H^{1.25502}$	$V=0.0000489823\times DBH^{1.60450}\times H^{1.25502}$	$V=0.0000489823\times DBH^{1.60450}\times H^{1.25502}$	$V=0.0000489823\times DBH^{1.60450}\times H^{1.25502}$
Ash Plantation	ASH-P	*Fraxinus griffithii*	$V=0.0000772\times DBH^{1.8780277}\times H^{0.8124601}$	$V=0.0000772\times DBH^{1.8780277}\times H^{0.8124601}$	$V=0.0000772\times DBH^{1.8780277}\times H^{0.8124601}$	$V=0.0000772\times DBH^{1.8780277}\times H^{0.8124601}$	$V=0.0000772\times DBH^{1.8780277}\times H^{0.8124601}$
Japanese Elm Plantation	JE-P	*Zelkova serrata*	$V=0.0000834\times DBH^{1.8761885}\times H^{0.805827}$	$V=0.0000834\times DBH^{1.8761885}\times H^{0.805827}$	$V=0.0000834\times DBH^{1.8761885}\times H^{0.805827}$	$V=0.0000834\times DBH^{1.8761885}\times H^{0.805827}$	$V=0.0000834\times DBH^{1.8761885}\times H^{0.805827}$
Sapphire Dragon Tree Plantation	SDT-P	*Paulownia kawakamii*	$V=0.0000464\times DBH^{1.53578}\times H^{1.50657}$	$V=0.0000464\times DBH^{1.53578}\times H^{1.50657}$	$V=0.0000464\times DBH^{1.53578}\times H^{1.50657}$	$V=0.0000464\times DBH^{1.53578}\times H^{1.50657}$	$V=0.0000464\times DBH^{1.53578}\times H^{1.50657}$
Other Broadleaved Plantation	O-B-P	Mixed Broadleaf	$V=0.0000464\times DBH^{1.53578}\times H^{1.50657}$	$V=0.0000464\times DBH^{1.53578}\times H^{1.50657}$	$V=0.0000464\times DBH^{1.53578}\times H^{1.50657}$	$V=0.0000464\times DBH^{1.53578}\times H^{1.50657}$	$V=0.0000464\times DBH^{1.53578}\times H^{1.50657}$
Mixed Broadleaved Plantation	MB-P	Mixed Broadleaf	$V=0.0000464\times DBH^{1.53578}\times H^{1.50657}$	$V=0.0000464\times DBH^{1.53578}\times H^{1.50657}$	$V=0.0000464\times DBH^{1.53578}\times H^{1.50657}$	$V=0.0000464\times DBH^{1.53578}\times H^{1.50657}$	$V=0.0000464\times DBH^{1.53578}\times H^{1.50657}$
Private Broadleaved Plantation	P-B-P	Mixed Broadleaf	$V=0.0000464\times DBH^{1.53578}\times H^{1.50657}$	$V=0.0000464\times DBH^{1.53578}\times H^{1.50657}$	$V=0.0000464\times DBH^{1.53578}\times H^{1.50657}$	$V=0.0000464\times DBH^{1.53578}\times H^{1.50657}$	$V=0.0000464\times DBH^{1.53578}\times H^{1.50657}$
New Broadleaved Plantation	NEW-B-P	Mixed Broadleaf	$V=0.0000464\times DBH^{1.53578}\times H^{1.50657}$	$V=0.0000464\times DBH^{1.53578}\times H^{1.50657}$	$V=0.0000464\times DBH^{1.53578}\times H^{1.50657}$	$V=0.0000464\times DBH^{1.53578}\times H^{1.50657}$	$V=0.0000464\times DBH^{1.53578}\times H^{1.50657}$
Natural Makino Bamboo Forest	MAK-BAM-NF	*Phyllostachys makinoi*	$V=(26.032\times DBH^{1.5777}\times H^{1.1237})\times 10^{-6}$	$V=(26.032\times DBH^{1.5777}\times H^{1.1237})\times 10^{-6}$	$V=(26.032\times DBH^{1.5777}\times H^{1.1237})\times 10^{-6}$	$V=(26.032\times DBH^{1.5777}\times H^{1.1237})\times 10^{-6}$	$V=(26.032\times DBH^{1.5777}\times H^{1.1237})\times 10^{-6}$
Natural Moso Bamboo Forest	MOS-BAM-NF	*Phyllostachys makinoi*	$V=(26.032\times DBH^{1.5777}\times H^{1.1237})\times 10^{-6}$	$V=(26.032\times DBH^{1.5777}\times H^{1.1237})\times 10^{-6}$	$V=(26.032\times DBH^{1.5777}\times H^{1.1237})\times 10^{-6}$	$V=(26.032\times DBH^{1.5777}\times H^{1.1237})\times 10^{-6}$	$V=(26.032\times DBH^{1.5777}\times H^{1.1237})\times 10^{-6}$
Natural Taiwan Giant Bamboo Forest	TG-BAM-NF	*Phyllostachys makinoi*	$V=(26.032\times DBH^{1.5777}\times H^{1.1237})\times 10^{-6}$	$V=(26.032\times DBH^{1.5777}\times H^{1.1237})\times 10^{-6}$	$V=(26.032\times DBH^{1.5777}\times H^{1.1237})\times 10^{-6}$	$V=(26.032\times DBH^{1.5777}\times H^{1.1237})\times 10^{-6}$	$V=(26.032\times DBH^{1.5777}\times H^{1.1237})\times 10^{-6}$
Natural Thorny Bamboo Forest	THO-BAM-NF	*Phyllostachys makinoi*	$V=(26.032\times DBH^{1.5777}\times H^{1.1237})\times 10^{-6}$	$V=(26.032\times DBH^{1.5777}\times H^{1.1237})\times 10^{-6}$	$V=(26.032\times DBH^{1.5777}\times H^{1.1237})\times 10^{-6}$	$V=(26.032\times DBH^{1.5777}\times H^{1.1237})\times 10^{-6}$	$V=(26.032\times DBH^{1.5777}\times H^{1.1237})\times 10^{-6}$
Natural Bambusa Forest	BAM-BAM-NF	*Phyllostachys makinoi*	$V=(26.032\times DBH^{1.5777}\times H^{1.1237})\times 10^{-6}$	$V=(26.032\times DBH^{1.5777}\times H^{1.1237})\times 10^{-6}$	$V=(26.032\times DBH^{1.5777}\times H^{1.1237})\times 10^{-6}$	$V=(26.032\times DBH^{1.5777}\times H^{1.1237})\times 10^{-6}$	$V=(26.032\times DBH^{1.5777}\times H^{1.1237})\times 10^{-6}$
Other Natural Bamboo Forest	O-BAM-NF	*Phyllostachys makinoi*	$V=(26.032\times DBH^{1.5777}\times H^{1.1237})\times 10^{-6}$	$V=(26.032\times DBH^{1.5777}\times H^{1.1237})\times 10^{-6}$	$V=(26.032\times DBH^{1.5777}\times H^{1.1237})\times 10^{-6}$	$V=(26.032\times DBH^{1.5777}\times H^{1.1237})\times 10^{-6}$	$V=(26.032\times DBH^{1.5777}\times H^{1.1237})\times 10^{-6}$
Natural Fargesia Forest	FAR-BAM-NF	*Phyllostachys makinoi*	$V=(26.032\times DBH^{1.5777}\times H^{1.1237})\times 10^{-6}$	$V=(26.032\times DBH^{1.5777}\times H^{1.1237})\times 10^{-6}$	$V=(26.032\times DBH^{1.5777}\times H^{1.1237})\times 10^{-6}$	$V=(26.032\times DBH^{1.5777}\times H^{1.1237})\times 10^{-6}$	$V=(26.032\times DBH^{1.5777}\times H^{1.1237})\times 10^{-6}$
Makino Bamboo Plantation	MAK-BAM-P	*Phyllostachys makinoi*	$V=(26.032\times DBH^{1.5777}\times H^{1.1237})\times 10^{-6}$	$V=(26.032\times DBH^{1.5777}\times H^{1.1237})\times 10^{-6}$	$V=(26.032\times DBH^{1.5777}\times H^{1.1237})\times 10^{-6}$	$V=(26.032\times DBH^{1.5777}\times H^{1.1237})\times 10^{-6}$	$V=(26.032\times DBH^{1.5777}\times H^{1.1237})\times 10^{-6}$

Moso Bamboo Plantation	MOS-BAM-P	*Phyllostachys makinoi*	$V=(26.032\times DBH^{1.5777}\times H^{1.1237})\times10^{-6}$	$V=(26.032\times DBH^{1.5777}\times H^{1.1237})\times10^{-6}$	$V=(26.032\times DBH^{1.5777}\times H^{1.1237})\times10^{-6}$	$V=(26.032\times DBH^{1.5777}\times H^{1.1237})\times10^{-6}$	$V=(26.032\times DBH^{1.5777}\times H^{1.1237})\times10^{-6}$
Taiwan Giant Bamboo Plantation	TG-BAM-P	*Phyllostachys makinoi*	$V=(26.032\times DBH^{1.5777}\times H^{1.1237})\times10^{-6}$	$V=(26.032\times DBH^{1.5777}\times H^{1.1237})\times10^{-6}$	$V=(26.032\times DBH^{1.5777}\times H^{1.1237})\times10^{-6}$	$V=(26.032\times DBH^{1.5777}\times H^{1.1237})\times10^{-6}$	$V=(26.032\times DBH^{1.5777}\times H^{1.1237})\times10^{-6}$
Thorny Bamboo Plantation	THO-BAM-P	*Phyllostachys makinoi*	$V=(26.032\times DBH^{1.5777}\times H^{1.1237})\times10^{-6}$	$V=(26.032\times DBH^{1.5777}\times H^{1.1237})\times10^{-6}$	$V=(26.032\times DBH^{1.5777}\times H^{1.1237})\times10^{-6}$	$V=(26.032\times DBH^{1.5777}\times H^{1.1237})\times10^{-6}$	$V=(26.032\times DBH^{1.5777}\times H^{1.1237})\times10^{-6}$
Bambusa Plantation	BAM-BAM-P	*Phyllostachys makinoi*	$V=(26.032\times DBH^{1.5777}\times H^{1.1237})\times10^{-6}$	$V=(26.032\times DBH^{1.5777}\times H^{1.1237})\times10^{-6}$	$V=(26.032\times DBH^{1.5777}\times H^{1.1237})\times10^{-6}$	$V=(26.032\times DBH^{1.5777}\times H^{1.1237})\times10^{-6}$	$V=(26.032\times DBH^{1.5777}\times H^{1.1237})\times10^{-6}$
Other Bamboo Plantation	O-BAM-P	*Phyllostachys makinoi*	$V=(26.032\times DBH^{1.5777}\times H^{1.1237})\times10^{-6}$	$V=(26.032\times DBH^{1.5777}\times H^{1.1237})\times10^{-6}$	$V=(26.032\times DBH^{1.5777}\times H^{1.1237})\times10^{-6}$	$V=(26.032\times DBH^{1.5777}\times H^{1.1237})\times10^{-6}$	$V=(26.032\times DBH^{1.5777}\times H^{1.1237})\times10^{-6}$
Private Bamboo Plantation	P-BAM-P	*Phyllostachys makinoi*	$V=(26.032\times DBH^{1.5777}\times H^{1.1237})\times10^{-6}$	$V=(26.032\times DBH^{1.5777}\times H^{1.1237})\times10^{-6}$	$V=(26.032\times DBH^{1.5777}\times H^{1.1237})\times10^{-6}$	$V=(26.032\times DBH^{1.5777}\times H^{1.1237})\times10^{-6}$	$V=(26.032\times DBH^{1.5777}\times H^{1.1237})\times10^{-6}$
Natural Coniferous and Broadleaved Mixed Forest	M-CB-NF	Mixed Broadleaf	$V=0.0000464\times DBH^{1.53578}\times H^{1.50657}$	$V=0.0000464\times DBH^{1.53578}\times H^{1.50657}$	$V=0.0000464\times DBH^{1.53578}\times H^{1.50657}$	$V=0.0000464\times DBH^{1.53578}\times H^{1.50657}$	$V=0.0000464\times DBH^{1.53578}\times H^{1.50657}$
		Mixed Conifer	$V=0.0000625\times DBH^{1.77924}\times H^{1.05866}$	$V=0.0000625\times DBH^{1.77924}\times H^{1.05866}$	$V=0.0000625\times DBH^{1.77924}\times H^{1.05866}$	$V=0.0000625\times DBH^{1.77924}\times H^{1.05866}$	$V=0.0000625\times DBH^{1.77924}\times H^{1.05866}$
Natural Bamboo and Coniferous Mixed Forest	M-BAMC-NF	*Chamaecyparis formosensis*	$V=0.00010092\times DBH^{1.54106}\times H^{1.155141}$	$V=0.00010092\times DBH^{1.54106}\times H^{1.155141}$	$V=-0.00324-0.00022\times DBH+0.00090\times H+0.00004\times DBH^2\times H-0.00001\times H^2$	$V=-0.00324-0.00022\times DBH+0.00090\times H+0.00004\times DBH^2\times H-0.00001\times H^2$	$V=-0.00324-0.00022\times DBH+0.00090\times H+0.00004\times DBH^2\times H-0.00001\times H^2$
		Chamaecyparis taiwanensis	$V=1.1439-0.0455\times DBH+0.0014\times DBH^2$	$V=1.1439-0.0455\times DBH+0.0014\times DBH^2$	$V=0.0000944\times DBH^{1.9947405}\times H^{0.659691}$	$V=0.0000944\times DBH^{1.9947405}\times H^{0.659691}$	$V=0.0000944\times DBH^{1.9947405}\times H^{0.659691}$
		Abies kawakamii	$V=-0.5066+0.005367\times DBH+0.000696\times DBH^2$	$V=-0.5066+0.005367\times DBH+0.000696\times DBH^2$	$V=-0.735678+0.006270\times DBH+0.000828\times DBH^2$	$V=-0.694737+0.006194\times DBH+0.000834\times DBH^2$	$V=-0.735678+0.006270\times DBH+0.000828\times DBH^2$
		Tsuga chinensis	$V=-0.1795-0.004396\times DBH+0.000695\times DBH^2$	$V=-0.1795-0.004396\times DBH+0.000695\times DBH^2$	$V=0.7295-0.035418\times DBH+0.0008846\times DBH^2$	$V=0.7847-0.03527\times DBH+0.00088\times DBH^2$	$V=0.7295-0.035418\times DBH+0.0008846\times DBH^2$
		Chamaecyparis taiwanensis	$V=1.1439-0.0455\times DBH+0.0014\times DBH^2$	$V=1.1439-0.0455\times DBH+0.0014\times DBH^2$	$V=0.0000944\times DBH^{1.9947405}\times H^{0.659691}$	$V=0.0000944\times DBH^{1.9947405}\times H^{0.659691}$	$V=0.0000944\times DBH^{1.9947405}\times H^{0.659691}$
		Chamaecyparis formosensis	$V=0.00010092\times DBH^{1.54106}\times H^{1.155141}$	$V=0.00010092\times DBH^{1.54106}\times H^{1.155141}$	$V=-0.00324-0.00022\times DBH+0.00090\times H+0.00004\times DBH^2\times H-0.00001\times H^2$	$V=-0.00324-0.00022\times DBH+0.00090\times H+0.00004\times DBH^2\times H-0.00001\times H^2$	$V=-0.00324-0.00022\times DBH+0.00090\times H+0.00004\times DBH^2\times H-0.00001\times H^2$
		Pinus taiwanensis	$V=0.0001547675\times DBH^{1.700988}\times H^{0.721114}$	$V=0.0001547675\times DBH^{1.700988}\times H^{0.721114}$	$V=0.0001547675\times DBH^{1.700988}\times H^{0.721114}$	$V=0.0001547675\times DBH^{1.700988}\times H^{0.721114}$	$V=0.0001547675\times DBH^{1.700988}\times H^{0.721114}$
		Picea morrisonicola	$V=(-1.0731)+0.021053\times DBH+0.000797\times DBH^2$	$V=(-1.0731)+0.021053\times DBH+0.000797\times DBH^2$	$V=-0.1997-0.02171\times DBH+0.000783\times DBH^2$	$V=-1.0731+0.021053\times DBH+0.000797\times DBH^2$	$V=-0.1997-0.02171\times DBH+0.000783\times DBH^2$
		Mixed Conifer	$V=0.0000625\times DBH^{1.77924}\times H^{1.05866}$	$V=0.0000625\times DBH^{1.77924}\times H^{1.05866}$	$V=0.0000625\times DBH^{1.77924}\times H^{1.05866}$	$V=0.0000625\times DBH^{1.77924}\times H^{1.05866}$	$V=0.0000625\times DBH^{1.77924}\times H^{1.05866}$
		Phyllostachys makinoi	$V=(26.032\times DBH^{1.5777}\times H^{1.1237})\times10^{-6}$	$V=(26.032\times DBH^{1.5777}\times H^{1.1237})\times10^{-6}$	$V=(26.032\times DBH^{1.5777}\times H^{1.1237})\times10^{-6}$	$V=(26.032\times DBH^{1.5777}\times H^{1.1237})\times10^{-6}$	$V=(26.032\times DBH^{1.5777}\times H^{1.1237})\times10^{-6}$
Natural Bamboo and Broadleaved Mixed Forest	M-BAMB-NF	Mixed Broadleaf	$V=0.0000464\times DBH^{1.53578}\times H^{1.50657}$	$V=0.0000464\times DBH^{1.53578}\times H^{1.50657}$	$V=0.0000464\times DBH^{1.53578}\times H^{1.50657}$	$V=0.0000464\times DBH^{1.53578}\times H^{1.50657}$	$V=0.0000464\times DBH^{1.53578}\times H^{1.50657}$
		Phyllostachys makinoi	$V=(26.032\times DBH^{1.5777}\times H^{1.1237})\times10^{-6}$	$V=(26.032\times DBH^{1.5777}\times H^{1.1237})\times10^{-6}$	$V=(26.032\times DBH^{1.5777}\times H^{1.1237})\times10^{-6}$	$V=(26.032\times DBH^{1.5777}\times H^{1.1237})\times10^{-6}$	$V=(26.032\times DBH^{1.5777}\times H^{1.1237})\times10^{-6}$
Natural Bamboo, Coniferous and Broadleaved Mixed Forest	M-BAMCB-NF	Mixed Broadleaf	$V=0.0000464\times DBH^{1.53578}\times H^{1.50657}$	$V=0.0000464\times DBH^{1.53578}\times H^{1.50657}$	$V=0.0000464\times DBH^{1.53578}\times H^{1.50657}$	$V=0.0000464\times DBH^{1.53578}\times H^{1.50657}$	$V=0.0000464\times DBH^{1.53578}\times H^{1.50657}$
		Mixed Conifer	$V=0.0000625\times DBH^{1.77924}\times H^{1.05866}$	$V=0.0000625\times DBH^{1.77924}\times H^{1.05866}$	$V=0.0000625\times DBH^{1.77924}\times H^{1.05866}$	$V=0.0000625\times DBH^{1.77924}\times H^{1.05866}$	$V=0.0000625\times DBH^{1.77924}\times H^{1.05866}$
		Chamaecyparis taiwanensis	$V=1.1439-0.0455\times DBH+0.0014\times DBH^2$	$V=1.1439-0.0455\times DBH+0.0014\times DBH^2$	$V=0.0000944\times DBH^{1.9947405}\times H^{0.659691}$	$V=0.0000944\times DBH^{1.9947405}\times H^{0.659691}$	$V=0.0000944\times DBH^{1.9947405}\times H^{0.659691}$
		Chamaecyparis formosensis	$V=0.00010092\times DBH^{1.54106}\times H^{1.155141}$	$V=0.00010092\times DBH^{1.54106}\times H^{1.155141}$	$V=-0.00324-0.00022\times DBH+0.00090\times H+0.00004\times DBH^2\times H-0.00001\times H^2$	$V=-0.00324-0.00022\times DBH+0.00090\times H+0.00004\times DBH^2\times H-0.00001\times H^2$	$V=-0.00324-0.00022\times DBH+0.00090\times H+0.00004\times DBH^2\times H-0.00001\times H^2$
		Phyllostachys makinoi	$V=(26.032\times DBH^{1.5777}\times H^{1.1237})\times10^{-6}$	$V=(26.032\times DBH^{1.5777}\times H^{1.1237})\times10^{-6}$	$V=(26.032\times DBH^{1.5777}\times H^{1.1237})\times10^{-6}$	$V=(26.032\times DBH^{1.5777}\times H^{1.1237})\times10^{-6}$	$V=(26.032\times DBH^{1.5777}\times H^{1.1237})\times10^{-6}$
Coniferous and Broadleaved Mixed Plantation	M-CB-P	Mixed Broadleaf	$V=0.0000464\times DBH^{1.53578}\times H^{1.50657}$	$V=0.0000464\times DBH^{1.53578}\times H^{1.50657}$	$V=0.0000464\times DBH^{1.53578}\times H^{1.50657}$	$V=0.0000464\times DBH^{1.53578}\times H^{1.50657}$	$V=0.0000464\times DBH^{1.53578}\times H^{1.50657}$
		Mixed Conifer	$V=0.0000625\times DBH^{1.77924}\times H^{1.05866}$	$V=0.0000625\times DBH^{1.77924}\times H^{1.05866}$	$V=0.0000625\times DBH^{1.77924}\times H^{1.05866}$	$V=0.0000625\times DBH^{1.77924}\times H^{1.05866}$	$V=0.0000625\times DBH^{1.77924}\times H^{1.05866}$
Bamboo and Coniferous Mixed Plantation	M-BAMC-P	Mixed Conifer	$V=0.0000625\times DBH^{1.77924}\times H^{1.05866}$	$V=0.0000625\times DBH^{1.77924}\times H^{1.05866}$	$V=0.0000625\times DBH^{1.77924}\times H^{1.05866}$	$V=0.0000625\times DBH^{1.77924}\times H^{1.05866}$	$V=0.0000625\times DBH^{1.77924}\times H^{1.05866}$
		Phyllostachys makinoi	$V=(26.032\times DBH^{1.5777}\times H^{1.1237})\times10^{-6}$	$V=(26.032\times DBH^{1.5777}\times H^{1.1237})\times10^{-6}$	$V=(26.032\times DBH^{1.5777}\times H^{1.1237})\times10^{-6}$	$V=(26.032\times DBH^{1.5777}\times H^{1.1237})\times10^{-6}$	$V=(26.032\times DBH^{1.5777}\times H^{1.1237})\times10^{-6}$
Bamboo and Broadleaved Mixed Plantation	M-BAMB-P	Mixed Broadleaf	$V=0.0000464\times DBH^{1.53578}\times H^{1.50657}$	$V=0.0000464\times DBH^{1.53578}\times H^{1.50657}$	$V=0.0000464\times DBH^{1.53578}\times H^{1.50657}$	$V=0.0000464\times DBH^{1.53578}\times H^{1.50657}$	$V=0.0000464\times DBH^{1.53578}\times H^{1.50657}$
		Phyllostachys makinoi	$V=(26.032\times DBH^{1.5777}\times H^{1.1237})\times10^{-6}$	$V=(26.032\times DBH^{1.5777}\times H^{1.1237})\times10^{-6}$	$V=(26.032\times DBH^{1.5777}\times H^{1.1237})\times10^{-6}$	$V=(26.032\times DBH^{1.5777}\times H^{1.1237})\times10^{-6}$	$V=(26.032\times DBH^{1.5777}\times H^{1.1237})\times10^{-6}$
Bamboo, Coniferous and Broadleaved Mixed Plantation	M-BAMCB-P	Mixed Conifer	$V=0.0000625\times DBH^{1.77924}\times H^{1.05866}$	$V=0.0000625\times DBH^{1.77924}\times H^{1.05866}$	$V=0.0000625\times DBH^{1.77924}\times H^{1.05866}$	$V=0.0000625\times DBH^{1.77924}\times H^{1.05866}$	$V=0.0000625\times DBH^{1.77924}\times H^{1.05866}$
		Mixed Broadleaf	$V=0.0000464\times DBHD^{1.53578}\times H^{1.50657}$	$V=0.0000464\times DBH^{1.53578}\times H^{1.50657}$	$V=0.0000464\times DBH^{1.53578}\times H^{1.50657}$	$V=0.0000464\times DBH^{1.53578}\times H^{1.50657}$	$V=0.0000464\times DBH^{1.53578}\times H^{1.50657}$
		Phyllostachys makinoi	$V=(26.032\times DBH^{1.5777}\times H^{1.1237})\times10^{-6}$	$V=(26.032\times DBH^{1.5777}\times H^{1.1237})\times10^{-6}$	$V=(26.032\times DBH^{1.5777}\times H^{1.1237})\times10^{-6}$	$V=(26.032\times DBH^{1.5777}\times H^{1.1237})\times10^{-6}$	$V=(26.032\times DBH^{1.5777}\times H^{1.1237})\times10^{-6}$
Private Coniferous and Broadleaved Mixed Plantation	P-M-CB-P	Mixed Conifer	$V=0.0000625\times DBH^{1.77924}\times H^{1.05866}$	$V=0.0000625\times DBH^{1.77924}\times H^{1.05866}$	$V=0.0000625\times DBH^{1.77924}\times H^{1.05866}$	$V=0.0000625\times DBH^{1.77924}\times H^{1.05866}$	$V=0.0000625\times DBH^{1.77924}\times H^{1.05866}$
		Mixed Broadleaf	$V=0.0000464\times DBH^{1.53578}\times H^{1.50657}$	$V=0.0000464\times DBH^{1.53578}\times H^{1.50657}$	$V=0.0000464\times DBH^{1.53578}\times H^{1.50657}$	$V=0.0000464\times DBH^{1.53578}\times H^{1.50657}$	$V=0.0000464\times DBH^{1.53578}\times H^{1.50657}$

Table S2 Weighting, above-ground biological expansion factor (*EF*), basic wood density (*D*), and carbon fraction (*CF*) of the referred species of 51 forest type

Forest type	Referred species	Weighting	*EF*	*D*	*CF*
FIR-NF	*Abies kawakamii**	1	1.60***	0.4	0.48
TSU-NF	*Tsuga chinensis*	1	1.27	0.4	0.49
CYP-NF	*Chamaecyparis taiwanensis**	0.5	1.36	0.4	0.48
	Chamaecyparis formosensis	0.5	1.36	0.3	0.49
PIN-NF	*Pinus taiwanensis*	1	1.22	0.6	0.47
SPR-NF	*Picea morrisonicola*	1	1.44	0.5	0.47
O-C-NF	Mixed Conifer	1	1.27	0.4	0.48
CYP-P	*Chamaecyparis taiwanensis*/Averaged Conifer	1	1.36	0.4	0.48
PIN-P	*Pinus massoniana*	1	1.27	0.4	0.48
LF-P	*Cunninghamia konishii*	1	1.68***	0.3	0.48
TAI-P	*Taiwania cryptomerioides*	1	1.23	0.3	0.48
JC-P	*Cryptomeria japonica*	1	1.32	0.4	0.49
TIC-P	*Calocedrus formosana*	1	1.27	0.5	0.49
O-C-P	Mixed Conifer	1	1.27	0.4	0.48
MC-P	Mixed Conifer	1	1.27	0.4	0.48
P-C-P	Mixed Broadleaf	1	1.51**	0.6	0.47
NEW-C-P	Mixed Conifer	1	1.27	0.4	0.48
B-NF	Mixed Broadleaf	1	1.37	0.6	0.47
MB-NF	Mixed Broadleaf	1	1.37	0.6	0.47
ACA-P	*Acacia confusa*	1	1.38	0.8	0.47
SG-P	*Liquidambar formosana**	1	1.38	0.6	0.47
CAM-P	*Cinnamomum camphora*	1	1.20	0.4	0.47
ASH-P	*Fraxinus formosana*	1	1.38	0.7	0.47
JE-P	*Zelkova serrata*	1	1.27	0.7	0.48
SDT-P	*Paulownia kawakamii**	1	1.38	0.6	0.47
O-B-P	Mixed Broadleaf	1	1.51**	0.6	0.47
MB-P	Mixed Broadleaf	1	1.51**	0.6	0.47
P-B-P	Mixed Broadleaf	1	1.51**	0.6	0.47
NEW-B-P	Mixed Broadleaf	1	1.51**	0.6	0.47
MAK-BAM-NF	*Phyllostachys makinoi*	1	1.50***	0.5	0.48
MOS-BAM-NF	*Phyllostachys makinoi*	1	1.50***	0.5	0.48
TG-BAM-NF	*Phyllostachys makinoi*	1	1.50***	0.5	0.48
THO-BAM-NF	*Phyllostachys makinoi*	1	1.50***	0.5	0.48
BAM-BAM-NF	*Phyllostachys makinoi*	1	1.50***	0.5	0.48
O-BAM-NF	*Phyllostachys makinoi*	1	1.50***	0.5	0.48
FAR-BAM-NF	*Phyllostachys makinoi*	1	1.50***	0.5	0.48
MAK-BAM-P	*Phyllostachys makinoi*	1	1.50***	0.5	0.48
MOS-BAM-P	*Phyllostachys makinoi*	1	1.50***	0.5	0.48
TG-BAM-P	*Phyllostachys makinoi*	1	1.50***	0.5	0.48
THO-BAM-P	*Phyllostachys makinoi*	1	1.50***	0.5	0.48
BAM-BAM-P	*Phyllostachys makinoi*	1	1.50***	0.5	0.48
O-BAM-P	*Phyllostachys makinoi*	1	1.50***	0.5	0.48
P-BAM-P	*Phyllostachys makinoi*	1	1.50***	0.5	0.48
M-CB-NF	Mixed Broadleaf	0.25	1.37	0.6	0.47
	Mixed Conifer	0.25	1.27	0.4	0.48
	Chamaecyparis formosensis	0.25	1.36	0.3	0.49
	Chamaecyparis taiwanensis	0.25	1.36	0.4	0.48
M-BAMC-NF	*Abies kawakamii**	0.08	1.60***	0.4	0.48
	Tsuga chinensis	0.08	1.27	0.4	0.49
	*Chamaecyparis taiwanensis**	0.04	1.36	0.4	0.48
	Chamaecyparis formosensis	0.04	1.36	0.3	0.49
	Pinus taiwanensis	0.08	1.22	0.6	0.47
	Picea morrisonicola	0.08	1.44	0.5	0.47
	Mixed Conifer	0.08	1.27	0.4	0.48
	Phyllostachys makinoi	0.5	1.50***	0.5	0.48
M-BAMB-NF	Mixed Broadleaf	0.5	1.51**	0.6	0.47
	Phyllostachys makinoi	0.5	1.50***	0.5	0.48
M-BAMCB-NF	Mixed Broadleaf	0.125	1.51**	0.6	0.47
	Mixed Conifer	0.125	1.27	0.4	0.48
	*Chamaecyparis taiwanensis**	0.125	1.36	0.4	0.48
	Chamaecyparis formosensis	0.125	1.36	0.3	0.49
	Phyllostachys makinoi	0.5	1.50***	0.5	0.48
M-CB-P	Mixed Broadleaf	0.5	1.51**	0.6	0.47
	Mixed Conifer	0.5	1.27	0.4	0.48
M-BAMC-P	Mixed Conifer	0.5	1.27	0.4	0.48
	Phyllostachys makinoi	0.5	1.50***	0.5	0.48
M-BAMB-P	Mixed Broadleaf	0.5	1.51**	0.6	0.47
	Phyllostachys makinoi	0.5	1.50***	0.5	0.48
M-BAMCB-P	Mixed Conifer	0.25	1.27	0.4	0.48
	Mixed Broadleaf	0.25	1.51**	0.6	0.47
	Phyllostachys makinoi	0.5	1.50***	0.5	0.48
P-M-CB-P	Mixed Conifer	0.5	1.27	0.4	0.48
	Mixed Broadleaf	0.5	1.51**	0.6	0.47

*: *D* and *CF* were defined as the averaged value of all coniferous or broadleaved species in Lin et al. (2002b)
**: 1.37 if in subtropical mountain system ecological zone
***: Includes below-ground biomass

Table S3 Diameter at breast height (*DBH*), tree height (*H*), stand density (*N*), and coverage area (*A*) of 51 forest types

Forest type	*DBH* (cm)	*H* (m)	*N* (stem ha^{-1})	*A* (ha)
FIR-NF	26.0	10	1394	21500
TSU-NF	30.8	13	1108	54000
CYP-NF	30.1	11	845	49300
PIN-NF	27.9	14	1140	70300
SPR-NF	41.1	18	613	7100
O-C-NF	40.7	19	790	23200
CYP-P	18.6	10	1217	25200
PIN-P	16.6	13	1860	46200
LF-P	16.6	12	2263	30300
TAI-P	19.2	11	1363	5000
JC-P	28.1	17	1524	47000
TIC-P	16.6	12	1796	1300
O-C-P	23.3	17	1313	1700
MC-P	21.6	11	1403	45000
P-C-P	19.9	13	1619	4000
NEW-C-P	19.9	13	1619	200
B-NF	18.8	11	889	15600
MB-NF	21.6	11	1004	721300
ACA-P	16.1	10	2067	36600
SG-P	11.8	11	1650	4100
CAM-P	25.5	12	716	5800
ASH-P	12.9	11	1741	13300
JE-P	10.2	5	672	5600
SDT-P	19.1	13	956	8300
O-B-P	15.4	11	1490	13900
MB-P	16.7	10	1388	26200
P-B-P	18.7	11	1357	500700
NEW-B-P	15.9	10	1327	3400
MAK-BAM-NF	4.1	10	16723	10000
MOS-BAM-NF	8.4	13	5727	2000
TG-BAM-NF	10.9	14	9085	10400
THO-BAM-NF	4.1*	10*	16723*	2300
BAM-BAM-NF	4.1*	10*	16723*	100
O-BAM-NF	4.1*	10*	16723*	100
FAR-BAM-NF	4.1*	10*	16723*	18400
MAK-BAM-P	4.1*	10*	16723*	8200
MOS-BAM-P	8.4	13	5727	3000
TG-BAM-P	10.9	14	9085	7400
THO-BAM-P	4.1*	10*	16723*	11800
BAM-BAM-P	4.1*	10*	16723*	1000
O-BAM-P	4.1*	10*	16723*	400
P-BAM-P	4.1*	10*	16723*	21800
M-CB-NF	24.8	12	910	294100
M-BAMC-NF (tree)	32.8	14	790	100
M-BAMC-NF (bamboo)	4.1*	10*	16723*	
M-BAMB-NF (tree)	20.2	11	947	63700
M-BAMB-NF (bamboo)	4.1*	10*	16723*	
M-BAMCB-NF (tree)	24.8	12	910	800
M-BAMCB-NF (bamboo)	4.1*	10*	16723*	
M-CB-P	17.9	12	1279	39500
M-BAMC-P (tree)	21.6	11	1403	2100
M-BAMC-P (bamboo)	4.1*	10*	16723*	
M-BAMB-P (tree)	16.7	10	1388	25400
M-BAMB-P (bamboo)	4.1*	10*	16723*	
M-BAMCB-P (tree)	17.9	12	1279	5400
M-BAMCB-P (bamboo)	4.1*	10*	16723*	
P-M-CB-P	18.7	11	1357	100

*: parameters from *P. makinoi*

Table S4 Annual growth rate and annual mortality rate of 51 forest types

Forest type	Annual growth rate (%)	Annual mortality rate (%)
FIR-NF	0.97	0.12
TSU-NF	0.88	0.03
CYP-NF	0.98	0.09
PIN-NF	1.27	0.08
SPR-NF	0.97	0.12
O-C-NF	2.26	0.07
CYP-P	6.14	0.80
PIN-P	6.14	0.80
LF-P	6.14	0.80
TAI-P	6.14	0.80
JC-P	6.14	0.80
TIC-P	6.14	0.80
O-C-P	6.14	0.80
MC-P	6.14	0.80
P-C-P	6.14	0.80
NEW-C-P	6.14	0.80
B-NF	2.91	0.55
MB-NF	2.99	0.55
ACA-P	7.75	1.26
SG-P	7.75	1.26
CAM-P	7.75	1.26
ASH-P	7.75	1.26
JE-P	7.75	1.26
SDT-P	7.75	1.26
O-B-P	7.75	1.26
MB-P	7.75	1.26
P-B-P	7.75	1.26
NEW-B-P	7.75	1.26
MAK-BAM-NF	6.24	0.98
MOS-BAM-NF	6.24	0.98
TG-BAM-NF	6.24	0.98
THO-BAM-NF	6.24	0.98
BAM-BAM-NF	6.24	0.98
O-BAM-NF	6.24	0.98
FAR-BAM-NF	6.24	0.98
MAK-BAM-P	6.24	0.98
MOS-BAM-P	6.24	0.98
TG-BAM-P	6.24	0.98
THO-BAM-P	6.24	0.98
BAM-BAM-P	6.24	0.98
O-BAM-P	6.24	0.98
P-BAM-P	6.24	0.98
M-CB-NF	1.53	0.11
M-BAMC-NF	4.83	0.48
M-BAMB-NF	4.83	0.48
M-BAMCB-NF	4.83	0.48
M-CB-P	1.53	0.11
M-BAMC-P	4.83	0.48
M-BAMB-P	4.83	0.48
M-BAMCB-P	4.83	0.48
P-M-CB-P	1.53	0.11
